# Effects of dietary oyster peptide supplement on litter performance, immunological response of sows and growth performance of piglets

**DOI:** 10.3389/fvets.2025.1581041

**Published:** 2025-10-22

**Authors:** Xiaofeng Tian, Jialu Wang, Congcong Yao, Muyang Shao, Yatian Qi, Zihao Gao, Hongguo Wang, Wei Xia, Zhigang Wang, Chenyu Tao, Junjie Li

**Affiliations:** ^1^College of Animal Science and Technology, Hebei Agricultural University, Baoding, China; ^2^Tianjin Institute of Industrial Biotechnology, Chinese Academy of Sciences, Tianjin, China; ^3^Key Laboratory of Agro-Ecological Processes in Subtropical Region, Institute of Subtropical Agriculture, Chinese Academy of Sciences, Changsha, China; ^4^Ruijia Agricultural Development Co., Ltd., Langfang, China; ^5^Hebei Technology Innovation Center of Cattle and Sheep Embryo, Baoding, China

**Keywords:** oyster peptide, farrowing performance, late gestation period, lactation period, sow

## Abstract

**Introduction:**

Animal breeding and reproduction techniques has led to an increase in the number of offspring of sows. However, weak piglets and low survival rate of piglets occurs frequently. During pregnancy, the late gestation is the most crucial period for fetal weight gain. Oyster peptides (OPI) are regarded as functional active substances derived from oysters with significant nutritional and medicinal value, exhibiting considerable potential for application. It remains unclear whether oyster peptides can play an important role in the field of sow reproduction. In this study, the objective was to investigate the impact of incorporating OPI into the diets of sows during the late gestation and lactation phases on litter performance, immunological response of sows and the growth and development of piglets.

**Methods:**

100 sows were selected and were randomly divided into the control group (CON, basic diet) and the experimental group (OPI, supplemented with 2 mg/kg OPI in feed) with the feeding period from gestation day 90 to day 21 postpartum. First, litter performance, immunological response of sows and growth performance of piglets were analyzed; then, RNA-seq and q-PCR were used to detect the molecular mechanism of OPI.

**Results:**

The results demonstrated that the supplementation of OPI to the diets of sows in late gestation and lactation resulted in a significant increase in the number of healthy piglets and weaning survival rate (*p* ≤ 0.05); colostrum and placenta samples were collected during parturition, IgA, IgG and IgM in colostrum of sows also increased (*p* ≤ 0.05); expression levels of glucose transporter genes (*GLUT4*, *SLC2A1*), amino acid transporter genes (*SNAT1*, *LAT1*), and fatty acid transporter genes (*FABP3*) in the placenta were increased (*p* ≤ 0.05). The levels of pro-inflammatory factors (*IL-1*, *IL-6*, *IL-8*, *AVPI1* were significantly decreased in the placenta, whereas the levels of anti-inflammatory factors (*IL-10*), antioxidant genes (*SOD1*, *SOD2*, *GPX2*, *CAT*), and anti-apoptotic genes (*BCL2*, *BCL2L1*) were sign.

## 1 Introduction

The utilization of contemporary animal breeding and reproduction techniques has led to a substantial augmentation in the number of offspring per litter of sows. However, this development is concomitant with a series of challenges, including, but not limited to, low birth weight, a high incidence of weak piglets, a low piglet survival rate, high morbidity, and a decline in litter weight at weaning (1–3). The intake of colostrum and milk was lower in low-birth-weight piglets than in high-birth-weight piglets within the same litter (4). This had a detrimental effect on the growth, reproductive, and quality of life of the piglets (5). Researchers have confirmed that the material exchange between sows and fetuses intensifies during the late stages of gestation, and nutritional intake is crucial for fetal growth (6). Nutrient transfers of the conventional kind, such as amino acids and sugars, are inadequate in supplying fetal growth and development (7). Additionally, it has been shown that oxidative stress, a possible consequence of pregnancy, can lead to an increased incidence of low-birth-weight piglets (8). Therefore, adequate nutrition and antioxidant products supplement during the late gestation is essential for the health of the sow and the number of viable offspring (9).

Peptides have been demonstrated to be remarkably effective in promoting reproduction and enhancing litter performance, and they do not interfere with maternal protein uptake pathways, rendering them optimal nutritional supplements (10). Oyster peptide (OPI) has been demonstrated to possess functional activities that enhance immunity, antioxidant and anti-inflammatory properties (11). It has been reported that OPI exhibited high antioxidant activities and could promote cell growth (12–14). Furthermore, OPI effectively enhanced cell proliferation, differentiation, and mineralization in MC3T3-E1 cells (15). Other studies have indicated that OPI possesses anti-inflammatory properties and has the capacity to impede the expression of inflammatory cytokines, including TNF-α, IL-1β and IL-6, contributing to an enhancement in the body’s immune system (16, 17). We speculated that dietary OPI supplement to sows in the late gestation can reduce the oxidative stress level, enhance immunity, and strengthen the nutrient transport between sows and fetus. Therefore, the objective was to investigate the impact of incorporating OPI into the diets of sows during the late gestation and lactation phases on litter performance, immunological response of sows and the growth and development of piglets.

## 2 Materials and methods

### 2.1 Experimental materials

The OPI utilized in this experiment was obtained from the Biological Synthesis of Nutritional Resources Research Group, Tianjin Institute of Industrial Biotechnology, Chinese Academy of Sciences (18). During application, it was added at the recommended dosage to ensure the accuracy and standardization of the experimental conditions.

### 2.2 Experimental animals and design

This experiment was performed in September 2023 at Ruijia Agricultural Development Co., Ltd., Yongqing County, Langfang, Hebei Province, China. A total of 100 Canadian Landrace × Yorkshire sows with healthy hooves and healthy body conditions were selected on the 90th day of pregnancy. The selected sows were randomly divided into two groups: the control group (CON group) was fed a basic diet, while the experimental group received the basal diet supplemented with 2 mg/kg of OPI. The feeding period was from 90 days of pregnancy to 21 days post-farrowing, with 50 sows in each group. Base diets for sows in pregnancy and lactation, respectively, were selected according to the nutritional requirements of different periods (Table 1).

**Table 1:** Composition and nutrient levels of basal diets for gestating and lactating sows.

Basal diet for gestating sows	Value (%)	Basal diet for lactating sows	Value (%)
Ingredient composition (%)			
Corn	53	Corn	61.6
Wheat bran	12.5	Low-protein soybean meal (43%)	20.5
Low-protein soybean meal (43%)	10.8	Soybean hulls	4.3
Secondary flour (ash 3.5%)	10	4% lactating sow premix^b^	4
Rice bran meal	5	Full-fat rice bran	3
4% gestating sow premix^a^	4	Rice bran meal	2.4
Soybean hulls	3	Soybean oil	2
Bentonite	1	Imported fishmeal (Peru)	1
Soybean oil	0.5	Bentonite	1
Imported mycotoxin adsorbent	0.2	Imported mycotoxin adsorbent	0.2
Nutrient composition			
Crude protein (%)	14.16	Crude protein (%)	16
Crude fat (%)	3.45	Crude fat (%)	5.12
Crude fiber (%)	5.57	Crude fiber (%)	4.5
Digestible energy (kcal/kg)	2947.87	Digestible energy (kcal/kg)	3278.00
Net energy (kcal/kg)	2122.19	Net energy (kcal/kg)	2332.01
Lysine (%)	0.80	Lysine (%)	1.04
Calcium (%)	0.78	Calcium (%)	0.96
Phosphorus (%)	0.35	Phosphorus (%)	0.41

a Each kilogram of gestation sow premix contains trace elements, glucose, sodium chloride, and vitamins, with a sodium selenite content of 0.04%.

b Each kilogram of lactation sow premix contains trace elements, glucose, sodium chloride, and vitamins, with a sodium selenite content of 0.01%.

### 2.3 Feeding management

Prior to farrowing, the sows in each group were kept in pens. One week ante partum, the animals were moved into the farrowing unit and all allocated to individual farrowing pens. The environment was kept clean and dry, with regular ventilation, lighting and temperature control at 25 °C, and disinfection procedures carried out regularly. The pregnant sows were fed twice a day at 5:00 and 16:30, with each feeding event involving the provision of 1.5 kg of feed. On the day of farrowing, the pregnant sows were provided with a reduced amount of feed (1.2 kg/meal, twice a day), and feeding was then gradually resumed 1–2 days after giving birth (1.3 kg/meal, twice a day). The nursing sows were fed three times a day at 5:00, 10:30, and 16:30, with each feeding consisting of 2 kg. All sows had unrestricted access to clean drinking water to maintain their normal physiological functions.

### 2.4 Sample collection

During parturition, seven sows per group were randomly selected for sample collection. During sow farrowing, umbilical cords were tied with a short silk line and each piglet was marked with a numbered tag to match the individual piglets with their placentae. After placental expulsion and weight recording, the placentae were collected and snap-frozen in liquid nitrogen (3 to 4 cm from the cord insertion point) and then stored at −80 °C until analysis.

Colostrum samples were taken when the first piglet was expelled. Before colostrum sampling, the entire visible udder was cleaned with soap and water and dried with paper. Subsequently, colostrum was collected through manual milking by massaging evenly, and then stored at −80 °C until analysis (19).

### 2.5 Data measurement of sows and piglets

On delivery day, the litter size, numbers of born alive, stillborn piglets, mummified fetuses, and individual piglet weight of each sow were recorded. The weak and healthy piglets were identified by the birth weight. Birth weight of healthy piglet was more than 900 g, while birth weight of weak piglet was less than 900 g (20). On 7, 14, and 21 days in lactation period, the individual piglet weight was recorded. The survival rate was calculated by dividing the number of live piglets by the total number of piglets born.

### 2.6 Analysis of immunoglobulin levels

The immunoglobulin (IgA, IgE, IgG, IgM) concentrations in the colostrum of farrowing sows was detected by using an enzyme-linked immunosorbent assay (ELISA). The assay procedure was conducted according to the instructions provided in the relevant porcine ELISA kit manual (Jingmei, China). The specific operation was as follows: firstly, the standard sample gradient dilution treatment was conducted, and the standard sample and the sample to be tested were added to the enzyme standard coated plate. The plate was then incubated at 37 °C for 30 min. It was subsequently washed five times by washing solution. Subsequently, the enzyme reagent was added and the plate was incubated at 37 °C for a further 30 min. The plate was then washed on five separate occasions. Finally, the color development solution was added, and the plate was incubated at 37 °C for 10 min. The absorbance at 450 nm was read with an UV spectrophotometer.

### 2.7 RNA sequencing

Three samples of placentas from the same sow were randomly selected in each group for transcriptomic analysis. Firstly, the total RNA of the samples was extracted using a Trizol kit (Biomed, China) and the concentration and quality of the total RNA were detected using the NanoDrop 2000 (Thermo, Waltham, MA, United States). After enriched mRNA was subsequently reverse transcribed to cDNA, and cDNA ends were repaired, purified, and amplified. The resulting cDNA libraries were used with Novaseq 6000 (Illumina, United States) to obtain quantification and annotation information for all genes.

For the analysis of RNA sequencing, first, the clean reads were obtained by removing reads containing adapter ploy-N and low-quality reads from the raw data (raw reads). Next, TopHat2 software was used to map reads to the pig genome (Sscrofa 11.1). Cuffquant and cuffnorm (v2.2.1) were used to estimate the expression levels of all the transcripts and analyze expression levels for mRNAs by calculating FPKM. The various differentially expression genes (DEGs) were selected between the 2 groups by using the DESeq2 R package when the adjusted FDR <0.05 and the absolute value of the log2 (fold change) was larger than 1.3. The volcano plot and heatmap of DEGs were clustered using TBtools (version 1.046). Gene Ontology (GO, http://geneontology.org/) enrichment analysis of the DEGs was performed to assess their biological significance using the GOseq R package.

### 2.8 Real-time quantitative PCR (RT-qPCR)

RNA samples were the same with those used in transcriptomic analysis. QRT-PCR with SYBR fluorescent dye (Biotium, Bay Area, CA, United States) was used to detect the mRNA expression levels of the genes in the cells, and the PCR conditions were as follows: 95 °C for 2 min followed by 40 cycles of 95 °C for 5 s and 60 °C for 30 s. Each experiment was repeated four times. Finally, gene expression was quantified using the 2^−ΔΔCT^ method, and GAPDH was selected as housekeeping gene. Primers used to amplify each gene are listed in Table 2.

**Table 2:** Primer sequences of pigs.

Primer name	Primer sequence (5′–3′)	Accession number
*GAPDH*	F: TGAAGGTCGGAGTGAACGGA	NM_001206359.1
	R: TGGGTGGAATCATACTGGAACA	
*GLUT4*	F: CCTGGGCCGATTGGTTATTG	KU672523.1
	R: CAGAATCCCGATGACGATGC	
SLC2A1	F: GCTCCTGGTCCTGTTCTTCATCTTC	AH004821.2
	R: CTCGGGTGTCTTGTCGCTTTGG	
*SLC7A1*	F: AACCCAGACATCTTTGCCGTGATC	NM_001012613.1
	R: CCATTATGAAGCCCAGGACCAGAA	
*SNAT1*	F: GCACTCGGACCTCCTTCACAAG	NP_001389503.1
	R: AGGATGACAGCGACAATGACAGC	
*LAT1*	F: TACTTGGTTCTGCTGGTGTCC	NM_001317081.1
	R: GTTGTGGGCTGTGTAAAGGTG	
*4F2HC*	F: CACTGACTCCTCCGACCTAC	EU587016.1
	R: TACTGCGTGACCAGGAAACT	
*SOD1*	F: CAGGTGCAGGTCCTCACTTCAATC	NM_001190422.1
	R: GTCACATTGCCCAGGTCTCCAAC	
*SOD2*	F: TTCTGGACAAATCTGAGCCCTAACG	NM_214127.2
	R: CGACGGATACAGCGGTCAACTTC	
*GPX2*	F: TGCAACCAATTTGGACATCAG	NM_001115136.1
	R: TTCACGTCACACTTCTGGATAAGG	
*GPX3*	F: AAACAGGAACCGGGAGACAA	NM_001115155.1
	R: AGGACAGGCGTTCTTCAGGAA	
*GPX4*	F: TCCATGCACGAATTCTCAGCCAAG	NM_214407.1
	R: TCATTGAGAGGCCACATTGGTGAC	
*CAT*	F: TGCCCATACTTCCCGTCC	NM_214301.2
	R: GGTCCAGGTTACCGTCAG	
*IL-1*	F: TCTGCCCTGTACCCCAACTG	NM_214055.1
	R: CCAGGAAGACGGGCTTTTG	
*IL-6*	F: TCAGTCCAGTCGCCTTCTCC	NM_214399.1
	R: GGCATTTGTGGTGGGGTTAG	
*IL-8*	F: AAATACGCATTCCACACCTTTCCAC	AB057440.1
	R: TGCTGTTGTTGTTGCTTCTCAGTTC	
*IL-10*	F: ATGGGCGACTTGTTGCTGAC	NM_214041.1
	R: CACAGGGCAGAAATTGATGACA	
*FABP3*	F: CCAACATGACCAAGCCTACCACA	NM_001099931.1
	R: ACAAGTTTGCCTCCATCCAGTGT	
*FATP*	F: GGAGTAGAGGGCAAAGCAGG	NM_001134255.1
	R: AGGTCTGGCGTGGGTCAAAG	
*BCL2*	F: AGGATTGTGGCCTTCTTTGAGTT	XM_021099602.1
	R: CGGTTCAGGTACTCAGTCATCCA	
*BCL2L1*	F: GGTACCGGAGGGCATTCAG	NM_214285.1
	R: ACAATGCGACCCCAGTTCAC	
*CXCL10*	F: GCTGCTGCTCCTGCTTCTAGTG	NM_001001861.2
	R: AGGTGAATTCCTTGCACGGTCTG	
*CCL4*	F: CCTGCTGCTTCACATACA	NM_213779.1
	R: TTCAGTTCCAAGTCATCCAT	
*DNAJB1*	F: CCAGACCTCCAACAACATT	NM_001244430.1
	R: CGGAAGAACCTGCTCAAG	
*FBLN7*	F: CAGGATGTGAACGAATGC	XM_005662277.3
	R: AGAGATAATGGAAGGAGATGG	
*SATB2*	F: GCCAACCAACTCTTCTGTA	XM_021076069.1
	R: CGATTGAATGCCACTCTTG	
*SGK2*	F: GATGGAAGGCTCTGATACTG	XM_021078166.1
	R: GGACCGAAGACCTCTGTT	
*IL1RN*	F: ACTGGAAGAGAAGATAGATGTG	NM_214262.1
	R: GAGCGGATGAAGGTGAAG	
*POF1B*	F: GTAGTGTATGAGCGTGTGA	XM_021080031.1
	R: GGTCTGTGGATATGAAGTGA	
*AVPI1*	F: AAACAGGAACCGGGAGACAA	XM_001929060.5
	R: AGGACAGGCGTTCTTCAGGAA	

### 2.9 Statistical analysis

The data analysis of this experiment was conducted using SPSS (Version 25.0, IBM Corporation, Armonk, NY, United States). First, all data were detected normally distributed. Independent sample *t*-test was used for the comparison of number and weight of piglets between two groups, while the chi-square test for the comparison of rate of healthy piglets and survival rate of live piglets between two groups. All results were expressed as the mean ± standard deviation. A significance level of *p* ≤ 0.05 was established to indicate a statistically significant difference.

## 3 Results

### 3.1 Effects of dietary supplementation of OPI during late pregnancy on the farrowing performance of sows

As demonstrated in Table 3, the supplementation of OPI of sows during the late gestation period resulted significant decrease in the number of weak piglets (2.02 ± 2.31 vs. 1.23 ± 1.39, *p* ≤ 0.05). Rate of healthy piglets was significantly promoted in OPI group (92.01 ± 8.79 vs. 87.10 ± 12.97, *p* ≤ 0.05).

**Table 3:** Effects of dietary supplementation of OPI during late pregnancy on the farrowing performance of sows.

Group	CON Group (*n* = 44)	OPI Group (*n* = 48)	*p*-value
Number of piglets born	15.27 ± 3.87	15.81 ± 3.62	0.49
Number of live piglets	14.02 ± 3.54	14.23 ± 3.01	0.76
Litter weight of live piglets	17.42 ± 4.24	18.21 ± 3.87	0.35
Average weight of live piglets	1.27 ± 0.25	1.30 ± 0.22	0.61
Number of stillborn piglets	1.00 ± 1.41	1.23 ± 1.52	0.46
Number of mummified fetuses	0.27 ± 0.82	0.35 ± 0.64	0.59
Number of weak piglets	2.02 ± 2.31^a^	1.23 ± 1.39^b^	0.05
Number of healthy piglets	12.00 ± 2.94	13.00 ± 2.65	0.09
Rate of healthy piglets/%	87.10 ± 12.97^a^	92.01 ± 8.79^b^	0.04

Different letters indicate significant differences (*p* ≤ 0.05).

### 3.2 Effect of OPI on growth performance of piglets during the suckling period

Dietary OPI supplementation of sows could significantly increase litter weight of live piglets at 7 days (29.45 ± 7.54 vs. 32.75 ± 6.77, *p* ≤ 0.05) (Table 4). As can be observed from Table 5, the number of live piglets at 14 days of the OPI group (10.61 ± 2.37 vs. 11.63 ± 1.85), litter weight of live piglets (43.98 ± 10.67 vs. 49.49 ± 10.57) and the survival rate of piglets at 14 days (77.81 ± 15.14 vs. 83.45 ± 11.91) were all significantly increased compared to the CON group (*p* ≤ 0.05). It could be found from Table 6 that OPI supplementation could significantly increase the number of live piglets weaned at 21 days (10.32 ± 2.35 vs. 11.44 ± 1.77, *p* ≤ 0.05), litter weight of live piglets at 21 days (59.22 ± 16.40 vs. 67.81 ± 14.46, *p* ≤ 0.05) and weaning survival rate (75.74 ± 15.69 vs. 82.22 ± 12.08, *p* ≤ 0.05) compared to the CON group (*p* ≤ 0.05).

**Table 4:** Effects of dietary oyster peptide supplementation in lactating sows on the growth performance of 7-day-old piglets.

Group	CON Group (n = 44)	OPI Group (n = 48)	*p*-value
Number of live piglets at 7 days	11.30 ± 2.53	12.08 ± 2.20	0.10
Litter weight of live piglets at 7 days	29.45 ± 7.54^a^	32.75 ± 6.77^b^	0.03
Average weight of live piglets at 7 days	2.65 ± 0.62	2.71 ± 0.34	0.56
Daily weight gain of live piglets at 7 days	0.20 ± 0.07	0.20 ± 0.04	0.66
Survival rate of piglets at 7 days	82.33 ± 13.80	86.33 ± 10.57	0.13

Different letters indicate significant differences (*p* ≤ 0.05).

**Table 5:** Effects of dietary oyster peptide supplementation in lactating sows on the growth performance of 14-day-old piglets.

Group	CON group (*n* = 44)	OPI group (*n* = 48)	*p*-value
Number of live piglets at 14 days	10.61 ± 2.37^a^	11.63 ± 1.85^b^	0.03
Litter weight of live piglets at 14 days	43.98 ± 10.67^a^	49.49 ± 10.57^b^	0.02
Average weight of live piglets at 14 days	4.16 ± 0.52	4.26 ± 0.66	0.42
Daily weight gain of live piglets at 14 days	0.21 ± 0.03	0.21 ± 0.05	0.50
Survival rate of piglets at 14 days	77.81 ± 15.14^a^	83.45 ± 11.91^b^	0.05

Different letters indicate significant differences (*p* ≤ 0.05).

**Table 6:** Effects of dietary oyster peptide supplementation in lactating sows on the growth performance of 21-day-old piglets.

Group	CON Group (*n* = 44)	OPI Group (*n* = 48)	*p*-value
Number of live piglets at 21 days	10.32 ± 2.35^a^	11.44 ± 1.77^b^	0.01
Litter weight of live piglets at 21 days	59.22 ± 16.40^a^	67.81 ± 14.46^b^	0.01
Average weight of live piglets at 21 days	5.75 ± 0.88	5.95 ± 0.97	0.31
Daily weight gain of live piglets at 21 days	0.21 ± 0.04	0.22 ± 0.04	0.32
Weaning survival rate at 21 days	75.74 ± 15.69^a^	82.22 ± 12.08^b^	0.03

Different letters indicate significant differences (*p* ≤ 0.05).

### 3.3 Effect of OPI on immunoglobulin levels in the colostrum of sows

**Figure 1:**
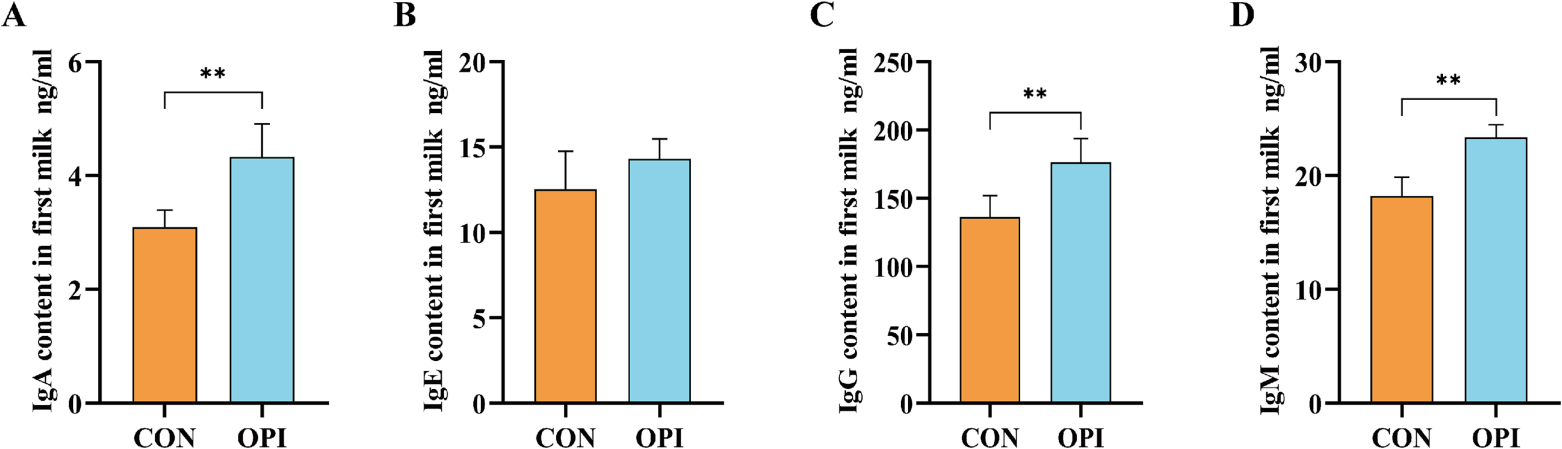
Effects of adding oyster peptide to the diet of sows in late pregnancy on the levels of immunoglobulin in sow’s colostrum. **(A)** The level of IgA in colostrum. **(B)** The level of IgE in colostrum. **(C)** The level of IgG in colostrum. **(D)** The level of IgM in colostrum. ^**^Represents significant difference at *p* ≤ 0.01.

As shown in Figure 1, the OPI supplementation could significantly increased the concentrations of IgA, IgG, and IgM in the colostrum of farrowing sows compared with the CON group (*p* ≤ 0.05).

### 3.4 Expression of transporter carriers, antioxidant and immune genes in placenta of sows supplemented with OPI

**Figure 2:**
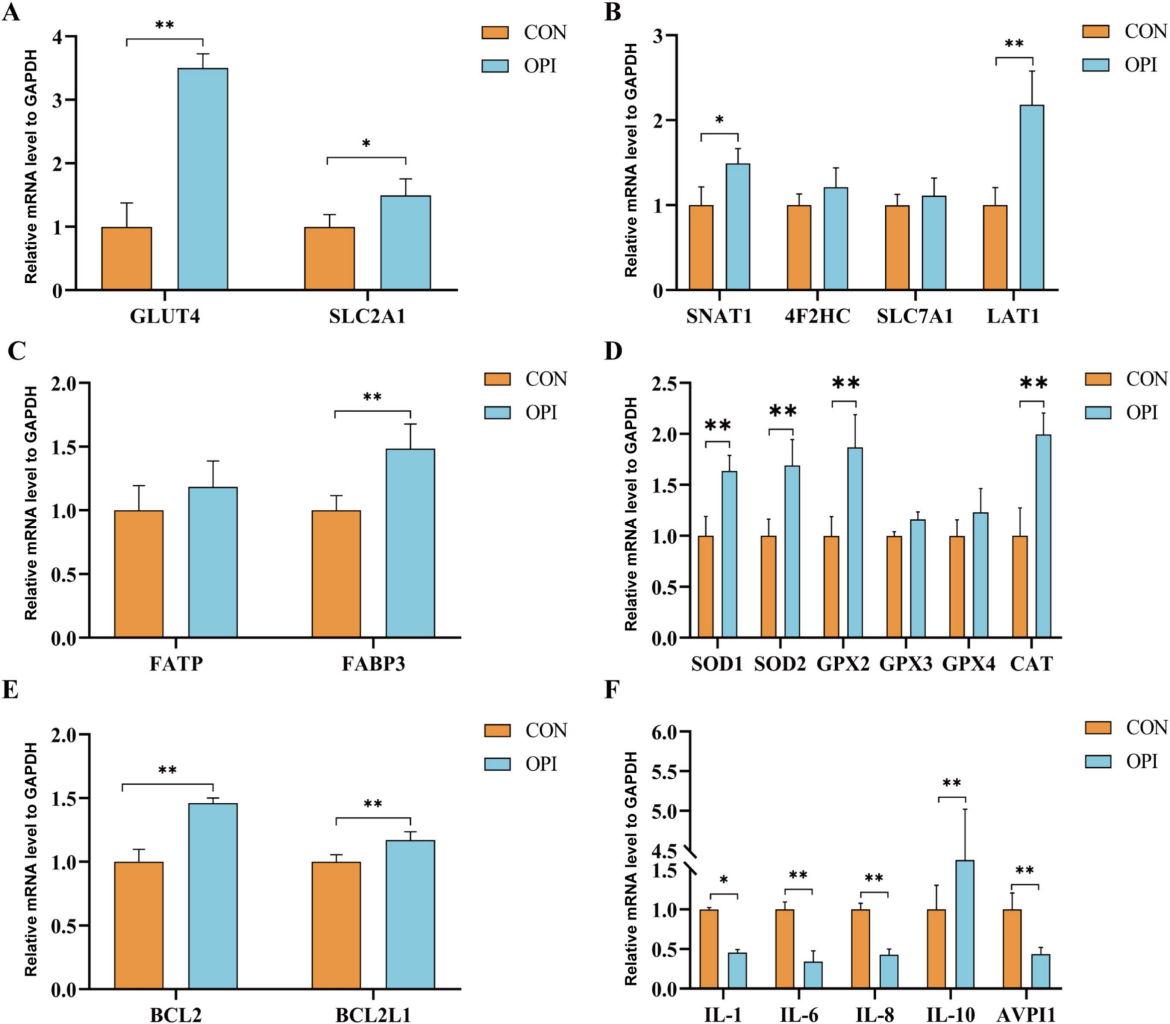
Effects of adding oyster peptide to the diet of sows in late pregnancy on the function of placenta. **(A)** The expression level of placental glucose related genes. **(B)** The expression level of placental amino-acid related genes. **(C)** The expression level of placental fatty -acid related genes. **(D)** The expression level of placental antioxidant-related genes. **(E)** The expression level of placental anti-apoptosis-related genes. **(F)** The expression levels of inflammatory cytokines within the placenta. ^*^Represents significant difference at *p* ≤ 0.05. ^**^Represents significant difference at *p* ≤ 0.01.

The expression levels of glucose transporter carriers (*GLUT4*, *SLC2A1*), amino acid transporter carriers (*SNAT1*, *LAT1*), fatty acid transporter carriers (*FABP3*) and antioxidant genes (*SOD1*, *SOD2*, *GPX2*, *CAT*) in the placenta was significantly increased compared to the CON group (Figures 2A–D, *p* ≤ 0.05). Anti-apoptotic genes (*BCL2*, *BCL2L1*) were also significantly higher expressed in the OPI group (Figure 2E, *p* ≤ 0.05). As shown in Figure 2F, the OPI supplementation significantly decreased the expression level of pro-inflammatory factors (*IL-1*, *IL-6*, *IL-8*, *AVPI1*) and significantly increased the expression levels of anti-inflammatory factors (*IL-10*) in sow placenta (*p* ≤ 0.05).

### 3.5 Transcriptomics analysis of placenta

**Figure 3:**
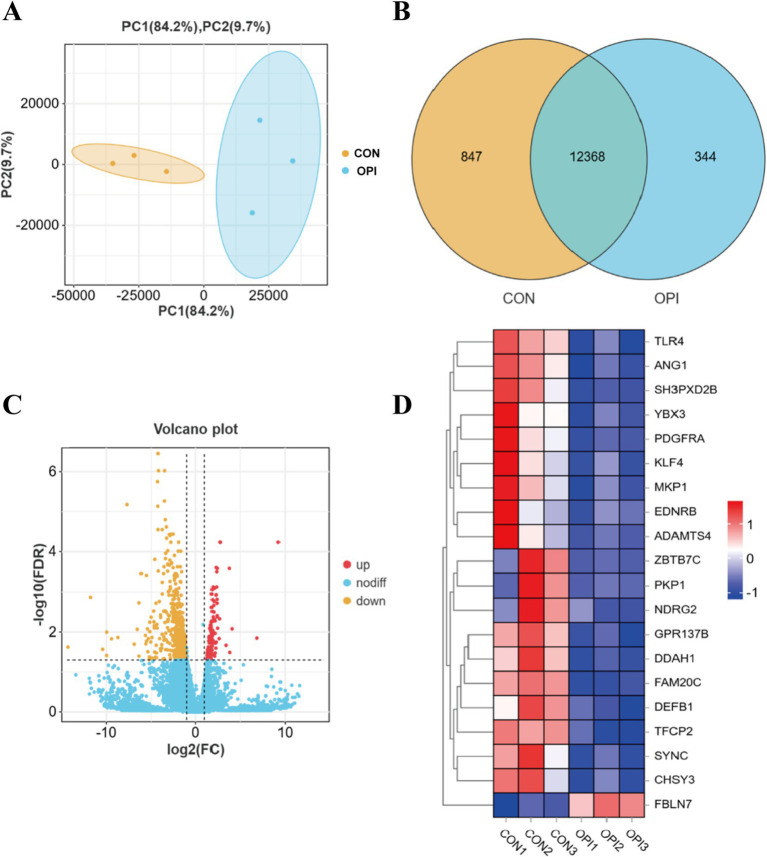
Analysis of differentially expressed genes (DEGs) in RNA-seq. **(A)** Principal component confidence diagram analysis of OPI and CON groups. **(B)** mRNAs expression statistics. **(C)** Volcano plot of differentially expressed genes (DEGs) between OPI and CON groups. **(D)** Heatmap of top 20 DEGs.

Based on the PCA analyses of transcriptomics, the profiles in the OPI group were distinct from those in the CON group (Figure 3A). A total of 13,215 known genes were identified in the CON group of this experiment, of which 847 were uniquely expressed. A total of 12,712 known genes were identified in the OPI group, of which 344 were uniquely expressed (Figure 3B). There was a total of 730 differentially expressed genes (DEGs) between the two groups, including 111 up-regulated genes and 619 down-regulated genes (Figure 3C) in OPI group compared to CON group. The heatmap demonstrated the top 20 DEGs (Figure 3D).

**Figure 4:**
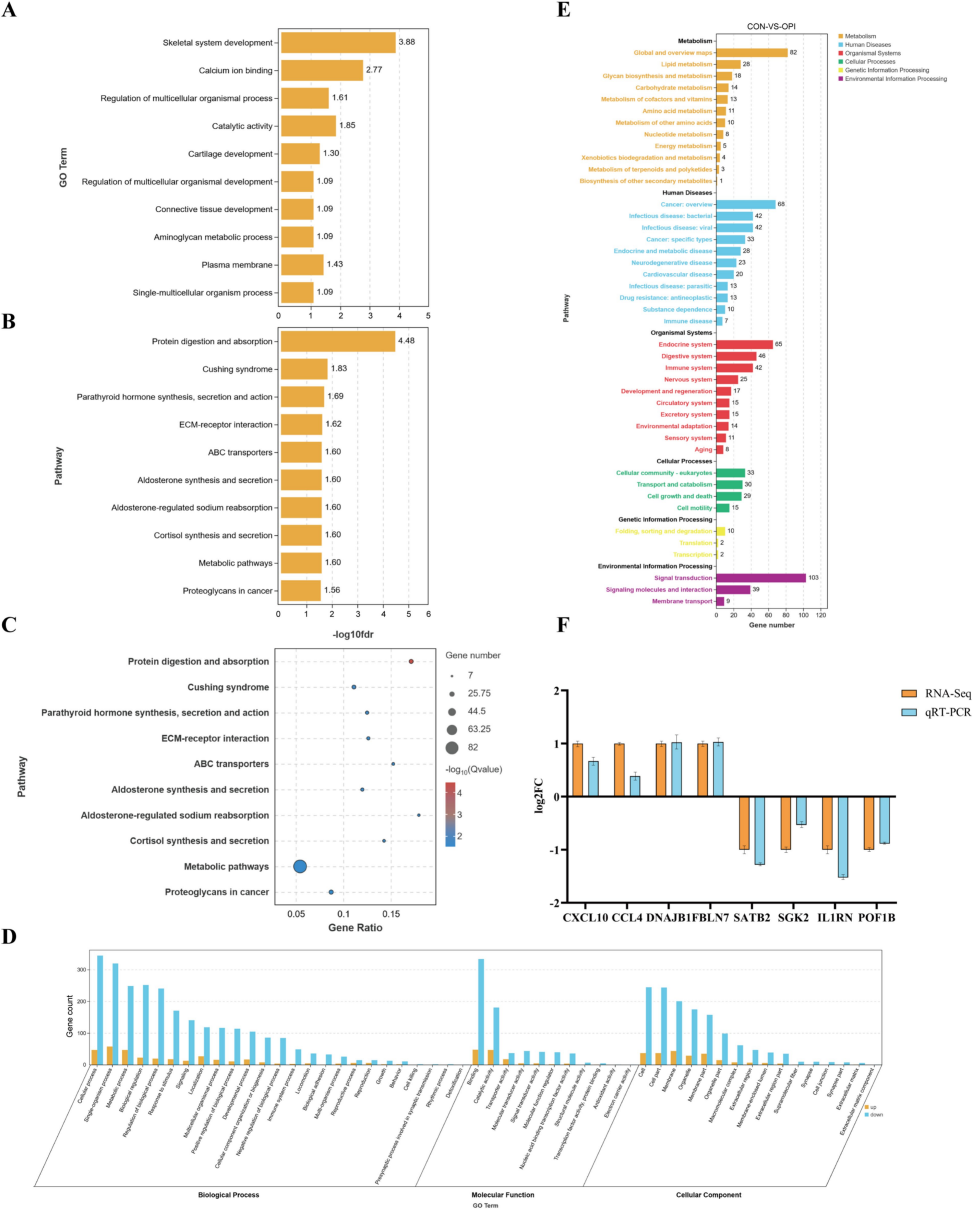
Enrichment analysis and verification of DEGs. **(A)** GO enrichment analysis of DEGs, value in *X*-axis represented the enrichment of GO terms. **(B)** KEGG enrichment analysis of DEGs, value in *X*-axis represented the enrichment of pathway. **(C)** KEGG significance bubble chart. **(D)** GO enrichment functional classification diagram. **(E)** KEGG quantity statistical chart. **(F)** Expression validation of 8 randomly selected DEGs by q-PCR. *Y*-axis represented the log2 FC (RNA-seq) or relative mRNA expression to GAPDH (q-PCR).

In order to further explore the molecular mechanism in the function of OPI, GO and KEGG analysis of DEGs were performed. GO analysis demonstrated that the DEGs were predominantly associated with skeletal system development, regulation of multicellular organismal process, connective tissue development and plasma membrane (Figure 4A). The majority of the DEGs were enriched in cellular process, metabolic process, biological regulation (biological processes); binding, transporter activity, molecular transducer activity, molecular function regulator (molecular functions) and composition of cell, membrane and macromolecular complex (cellular components) (Figure 4D).

KEGG enrichment analysis demonstrated that the DEGs were predominantly associated with metabolism signaling and cortisol synthesis, secretion, including metabolic pathways, protein digestion, parathyroid hormone synthesis and ABC protein transport (Figures 4B,C,E).

As demonstrated in Figure 4F, randomly selected eight DEGs (*CXCL10*, *CCL4*, *DNAJB1*, *FBLN7*, *SATB2*, *SGK2*, *IL1RN*, *POF1B*) were subjected to qRT-PCR for validation in the CON and OPI groups. There were similar directions of the fold change of relative expression level in qRT-PCR and RNA-seq of the genes. This result verified the reliability of the sequencing data.

## 4 Discussion

Fetus grows and develops fastest in sows in the late gestation period, with significant enhancements in energy, protein and fat (21). Previous research has revealed bioactive ingredient can enhance the growth performance, by improving digestive function, inflammation resistance and the enhancement of antioxidant properties (22, 23). Another study revealed that the supplementation of N-carbamoyl-aspartic dipeptide into the diet of sows resulted in an augmentation of the litter weight of piglets, in addition to an increase in the survival rate of the piglets (18). The OPI produced from pichia pastoris was used in this study. It is a mixture, and its purity is not measured by the content of peptides, but by its antioxidant performance. The mixture is abundant in protein, taurine, glycogen and other nutrients. Since the dosage of OPI was 2 mg/kg, the protein and other nutrients in it had very little impact on the nutritional components of the feed, thus OPI is more regarded as an active additive.

However, there was no research about OPI supplementation on the litter performance, immunological response in sows, the addition of OPI resulted in a significant increase in the number of healthy litters (birth weight of healthy piglet ≥900 g), the number of live weaned piglets, the weaning litter weight and the weaning survival rate in this study as we expected. The function of OPI, enhancing immunity, antioxidant and anti-inflammatory properties, showed in this study was consistent with the previous research (12, 13).

The placenta, an organ that connects the fetus to the mother, plays a crucial role in facilitating fetal nutritional intake (24). Ren’s study demonstrated that the incorporation of the active peptide CC34 led to a substantial augmentation in the relative expression of sheep amino acid transporter carriers (25). The addition of the honeybee antimicrobial peptide Api-PR19 was indicated by Wang (26) to significantly increase the expression levels of glucose and fatty acid transporter carriers. The results in this study demonstrated that OPI could promoted the expression of glucose (GLUT4, SLC2A1), amino acids (SNAT1, LAT1), and fatty acid (FABP3) transporter carriers in the placenta of sows. Concurrently, the number of healthy piglets increased, while the number of weak piglets decreased. Therefore, OPI could help improve the production performance of sows through its nutritional components and improving placental transport function.

During the late pregnancy period, the maternal immune system is reduced and vulnerable to inflammatory factors. Inflammation is a basic pathological process that occurs in the body as a defense response to various damaging factors (such as infection, trauma, foreign bodies, etc.) (27, 28). It has been reported that the immune system of piglets was shaped by the placenta during gestation and further developed through colostrum after birth (29–31). Hwang et al. (17) found that OPI alleviated the inflammatory response induced by lipopolysaccharide and significantly reduced the expression level of pro-inflammatory factors by inhibiting the NF-κB pathway. Qian et al. (16) have reached similar conclusions, asserting that OPIs possess anti-inflammatory properties and are capable of alleviating the oxidative stress caused by lipopolysaccharides on macrophages. As demonstrated by reports such as the above, it can be concluded that OPI exerts some degree of anti-inflammatory effects. Our findings also showed a significant increase of anti-inflammatory factors (*IL-10*) in the placental with OPI supplementation. Furthermore, there is a substantial increase in immunoglobulins in the colostrum of sows, thereby enhancing the immunity of piglets and increasing survival rates. This might be due to the fact that the overall immune level of sows has been improved after OPI supplementation, which led to an increase in the level of immunoglobulin in colostrum.

Oxidative stress becomes more pronounced during late gestation and lactation of sows and piglets and did not recover until after weaning (32). It is directly associated with a rise of stillbirths and weak piglets (33). It has been established that elevated ROS could induce autophagy, dysfunction and apoptosis in vascular endothelial cells, leading to impaired development of the placental vasculature system and resulting in intrauterine fetal developmental restriction (34). Wang et al. (35) observed that administration of OPI to mice resulted in a significant decrease in ROS and a significant increase in SOD and GSH-Px, thereby significantly enhancing the antioxidant properties of the mice. Yan et al. (13) indicated that OPI may exert antioxidant effects by binding to ROS and interacting with antioxidant enzymes. In this study, the addition of OPI could increase *SOD*, *GSH-Px* and *CAT* expression levels, which was consistent with the previous research. This demonstrates OPI enhances the antioxidant properties of the placenta in sows, and this may be an important reason for the increase of healthy piglets.

In order to investigate the mechanism of OPI, q-PCR and transcriptome of placenta were analyzed. The pro-inflammatory factors (*IL*-*1*, *IL-6*, *IL-8*) were decreased while expression of anti-apoptotic genes (*BCL2*, *BCL2L1*) were increased in OPI group. In addition, key genes associated with placental transport and anti-inflammatory were identified. These results provided potential mechanisms of OPI on sow reproductive performance at the mRNA level.

## 5 Conclusion

Dietary OPI in sows during late gestation and lactation is a promising way to improve litter performance, immunological response of sows and growth performance of piglets. OPI supplementation could increase the expression of antioxidant and nutrient transport gene in placental, which may lead to better piglet birth weight and more healthy piglets. Moreover, it could also enhance immune function both in sows and colostrum, strengthening piglet passive immunity, and improving weaning litter weight and survival rate.

## Data Availability

The RNA-seq data from this paper were deposited in the NCBI Sequence Read Archive (SRA) database under accession number PRJNA1344342.
